# Structure and Antioxidant Activity Relationships of Isoflavonoids from *Dalbergia parviflora*

**DOI:** 10.3390/molecules19022226

**Published:** 2014-02-20

**Authors:** Worrawat Promden, Orawan Monthakantirat, Kaoru Umehara, Hiroshi Noguchi, Wanchai De-Eknamkul

**Affiliations:** 1 Division of General Science, Faculty of Education, Buriram Rajabhat University, Buriram 31000, Thailand; E-Mail: p_worrawat@yahoo.com; 2 Department of Pharmacognosy and Botany, Faculty of Pharmaceutical Sciences, Chulalongkorn University, Bangkok 10330, Thailand; 3 Department of Pharmaceutical Chemistry, Faculty of Pharmaceutical Sciences, Khon Kaen University, Khon Kaen 40002, Thailand; E-Mail: oramon_o@yahoo.com; 4 School of Pharmaceutical Sciences, University of Shizuoka, Shizuoka 422-8526, Japan; E-Mails: umehara@mail.u-shizuoka-ken.ac.jp (K.U.); noguchi@u-shizuoka-ken.ac.jp (H.N.)

**Keywords:** antioxidant, isoflavonoids, *Dalbergia parviflora*, DPPH, ORAC, xanthine/xanthine oxidase assay, structure-activity relationship

## Abstract

The antioxidant activities of 24 isoflavonoids that were previously isolated as pure compounds from *Dalbergia parviflora* were evaluated using three different *in vitro* antioxidant-based assay systems: xanthine/xanthine oxidase (X/XO), ORAC, and DPPH. The isolates consisted of three subgroups, namely isoflavones, isoflavanones, and isoflavans, each of which appeared to have diversified substituents, and were thus ideal for the study of their structure-activity relationships (SARs). The SAR analysis was performed using the results obtained from both the inter-subgroup isoflavonoids with the same substitution pattern and the intra-subgroup compounds with different substitution patterns. The inter-subgroup comparison showed that the isoflavones exhibited the highest antioxidant activities based on all three assays. The intra-subgroup analysis showed that the additional presence of an OH group in Ring B at either R3′ or R5′ from the basic common structure of the R7-OH of Ring A and the R4′-OH (or -OMe) of Ring B greatly increased the antioxidant activities of all of the isoflavonoid subgroups and that other positions of OH and OMe substitutions exerted different effects on the activities depending on the subgroup and assay type. Therefore, based on the structural diversity of the isoflavonoids in *D. parviflora*, the present study provides the first clarification of the detailed antioxidant SARs of isoflavonoids.

## 1. Introduction

Natural flavonoids and isoflavonoids have been found to influence intercellular redox status, to interact with specific proteins in intracellular signalling pathways, and to have antioxidant properties [1,2]. Of these two groups, due to their structural diversity, the structure-activity relationships (SARs) of flavonoids have been well studied using various antioxidant activity assays [3,4,5]. It has been reported that the intensity of the antioxidant activity of a flavonoid strongly depends on its chemical structure, which is particularly influenced by the number and position of hydroxyl groups attached to the two aromatic rings [3,4,5,6]. In contrast, the SARs of isoflavonoids based on their antioxidant activity are scarcely known. This might be due to the limited number of known natural isoflavonoids [6,7], which are not sufficiently diversified for SAR studies. However, we recently found that the heartwood of *Dalbergia parviflora* is a rich source of isoflavonoids. Up to 30 isoflavonoids with a potent range of estrogenic-like activities have been isolated from this Thai folk medicine [8,9]. The plant has been used as a blood tonic and for the normalisation of menstrual cycles. Interestingly, the isolated isoflavonoids appear to have diversified structures that belong to the well-known subgroups of isoflavones, isoflavanones, and isoflavans [8,9].

Therefore, the objective of this study was to investigate the *in vitro* antioxidant activities of the library set of isoflavonoids isolated from *D. parviflora*. The compounds were tested using three common methods with different working principles: the DPPH radical scavenging activity, the xanthine oxidase free radical-generating system, and oxygen radical absorbance capacity (ORAC). The results of the SAR were then evaluated and are reported in this manuscript.

## 2. Results and Discussion

### 2.1. SAR of D. parviflora Isoflavonoids Based on Xanthine/Xanthine Oxidase Assay

In this study, superoxide radicals (•O_2_^−^) were generated using the xanthine/xanthine oxidase assay system (X/XO assay), which catalyses the chain reactions of hypoxanthine oxidation to xanthine and from xanthine to uric acid and hydrogen peroxide [10]. The superoxide radical scavenging activities of each pure isoflavonoid are shown in Table 1. It can be observed that most of the isoflavonoids exhibited high scavenging activity against superoxide radicals. The subgroup of isoflavones, particularly calycosin (5), khrinone B (4), and khrinone C (8), showed the highest activities with 50% radical scavenging concentration (SC_50_) values of 0.25, 0.60, and 0.67 µM, respectively. This group was followed by the subgroup of isoflavans, including *(3RS)*-3′-hydroxy-8-methoxy vestitol (24) and *(3R)*-vestitol (21), which showed SC_50_ values of 2.8 and 6.4 µM, respectively. In the subgroup of isoflavanones, *(3RS)*-kenusanone G (15) with an SC_50_ 8.6 µM was the only compound that showed a comparable level of inhibition. These potencies of the different subgroups were clearly confirmed by comparing the activities of compounds belonging to the three subgroups that contain the same substitution pattern. As shown in Table 2, isoflavone khrinone (8) appeared to be much more potent than the isoflavan *(3S)*-8-demethylduartin (22) and the isoflavanone *(3S)*-secundiflorol H (17).

**Table 1:** SAR of *D. parviflora* isoflavonoids based on the xanthine/xanthine oxidase assay.

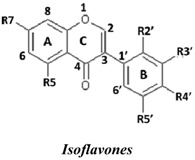	***No.***	***Isoflavones***	**R7**	**R5**	**R2′**	**R3′**	**R4′**	**R5′**	**SC_50_ (µM)**
	**5**	Calycosin	OH	H	H	OH	OMe	H	0.25 ± 0.05
	**4**	Khrinone B	OH	OH	OH	H	OMe	OH	0.60 ± 0.1
	**8**	Khrinone C	OH	OH	OMe	OH	OMe	H	0.64 ± 0.03
	**3**	Genistein	OH	OH	H	H	OH	H	9.0 ± 2.2
	**6**	3′-O-Methylorobol	OH	OH	H	OMe	OH	H	36.7 ± 7.2
	**7**	Cajanin	OMe	OH	OH	H	OH	H	54.3 ± 10.7
	**1**	Formononetin	OH	H	H	H	OMe	H	116.92 ± 15.6
	**2**	Biochanin A	OH	OH	H	H	OMe	H	203.3 ± 57.6
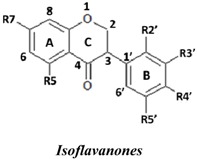	***No.***	***Isoflavanones***	**R7**	**R5**	**R2′**	**R3′**	**R4′**	**R5′**	**SC_50_ (µM)**
	**15**	3(*R,S*)-Kenusanone G	OH	OH	H	OH	OMe	H	8.6 ± 1.2
	**12**	3(*R*)-7,3′-Dihydroxy-4′-methoxyisoflavanone	OH	H	H	OH	OMe	H	27.9 ± 5.4
	**14**	Dalparvin B	OH	H	OH	OMe	OMe	H	30.5 ± 3.8
	**16**	3(*R,S*)-Violanone	OH	H	OMe	OH	OMe	H	43.7 ± 9.7
	**13**	3(*R,S*)-Dalparvin	OH	H	OMe	H	OMe	OH	48.2 ± 15.0
	**11**	3(*R,S*)-Onogenin	OH	H	OMe	H	OCH_2_O		56.9 ± 0.18
	**10**	3(*S*)-Sativanone	OH	H	OMe	H	OMe	H	59.3 ± 21.7
	**18**	3(*R*)-Dalparvin A	OH	OH	OMe	H	OH	OH	160.3 ± 54.4
	**17**	3(*S*)-Secundiflorol H	OH	OH	OMe	OH	OMe	H	247.2 ± 82.2
	**9**	3(*R,S*)-3′-O-Methyl-violanone	OH	H	OMe	OMe	OMe	H	-
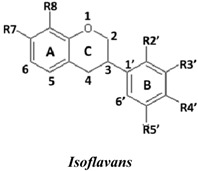	***No.***	***Isoflavans***	**R7**	**R8**	**R2′**	**R3′**	**R4′**	**R5′**	**SC_50_ (µM)**
	**24**	3(*R,S*)-3′-Hydroxy-8-methoxy vestitol	OH	OMe	OH	OH	OMe	H	2.8 ± 0.7
	**21**	(*3R*)-Vestitol	OH	H	OH	H	OMe	H	6.4 ± 0.1
	**23**	(*3R*)(+)-Mucronulatol	OH	H	OMe	OH	OMe	H	10.0 ± 3.6
	**22**	(*3S*)-8-Demethylduartin	OH	OH	OMe	OH	OMe	H	13.4 ± 3.6
	**20**	3(*R,S*)-Duartin	OH	OMe	OMe	OH	OMe	H	12.2 ± 4.2
	**19**	3(*R,S*)-Sativan	OH	H	OMe	H	OMe	H	12.8 ± 1.2

“-” no antioxidant activity was detected.

**Table 2:** Comparison of three isoflavonoids with the same substitution but belonging to different subgroups: the isoflavone khrinone C, the isoflavanone 3*(S)-*secundiflorol H, and the isoflavan 3*(S)-*8-demethylduartin.

Chemical Structures	X/XO assay, SC_50_ (µM)	ORAC assay, Trolox Equivalents (µM TE/10 µM isoflavonoid)	DPPH assay, SC_50_ (µM)
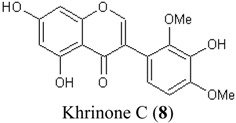	0.64 ± 0.03	43.5 ± 3.2	61.7 ± 4.5
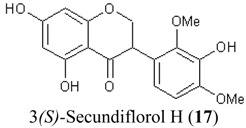	247.2 ± 82.2	27.4 ± 7.7	74.3 ± 4.2
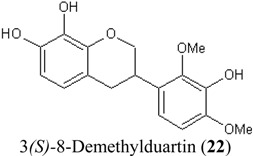	13.4 ± 3.6	27.0 ± 1.9	115.4 ± 4.3

A further SAR analysis revealed that these active isoflavonoids from all three subgroups have the following common substituent pattern: the presence of R7-OH in Ring A and the R4′-OMe in Ring B with either R3′-OH or R5′-OH (Table 1). However, the presence of R7-OH and R4′-OMe in the molecule with no other key substituents (*i.e.*, formononetin (**1**), SC_50_ = 117 µM), showed considerably lower activity, suggesting that these two substituents are not as important. The analysis of the position of the R2 of Ring B, interestingly, revealed that the presence of R2′-OMe can diminish the positive effect of the R3′-OH or R5′-OH substitution, especially in the subgroup of isoflavanones. As shown in Table 1, the structures of 3*(R,S)*-kenusanone G (**15**) and 3*(S)*-secundiflorol H (**17**) are different from each other only by the presence of R2′-H in the former and R2′-OMe in the latter, but their SC_50_ values were found to be highly different (8.6 and 247 µM, respectively).

Based on these results, it was concluded that the R3′-OH and R5′-OH substitutions in the isoflavonoids strongly promote the superoxide radical scavenging activities, as determined through the X/XO assay, whereas the R2′-OMe substitution reduces the effect. In the literature, very little has been reported regarding the SAR of isoflavonoids based on the X/XO assay. It has been shown that genistein (**3**) exhibits superoxide scavenging activity at a concentration range of 0.1 to 4.0 µM [11,12], that calycosin (**5**) at a concentration of 35 µM inhibits 52% of the xanthine oxidase activity, and that formononetin (**1**) has no effect at the same concentration [13]. Our results, however, showed much stronger activity for calycosin (**5**) than genistein (**3**). In terms of substituents, it has also been suggested that non-methylated R4′-OH is important for the inhibitory effect, as determined by the results obtained using the soybean isoflavonoids genistein (strong), daidzein (moderate), and biochanin A (no activity) [14]. Similarly, our results also showed the importance of the non-methylated R′4-OH of genistein (SC_50_ = 9.0 µM) which gave much stronger activity than the methylated R′4-OMe of biochanin A (SC_50_ = 203 µM).

### 2.2. SAR of D. parviflora Isoflavonoids Based on the ORAC Assay

The oxygen radical absorbance capacity (ORAC) assay is a method that directly measures the antioxidant activities of chain-breaking antioxidants against peroxyl radicals (ROO•) [15]. This assay is based on the inhibition of the peroxyl radical-induced oxidation initiated by the thermal decomposition of the azo compound 2,2′-azobis(2-amidinopropane) dihydrochloride (AAPH). The assay measures the loss of fluorescein fluorescence over time due to peroxyl radical formation. The subsequent addition of an antioxidant produces a more stable fluorescence signal, which depends on the capacity of the antioxidant. A water-soluble tocopherol analogue, namely Trolox (6-hydroxy-2,5,7,8-tetramethylchroman-2-carboxylic acid), has been used as a standard compound for comparing and determining the antioxidant capacity of a test compound [16,17].

In this study, the ORAC value of ascorbic acid was determined to be 5 µM Trolox equivalents per 10 µM ascorbic acid (Trolox is two-fold stronger than ascorbic acid). The analysis of the 24 pure isoflavonoids isolated from *D. parviflora* revealed interesting results, as illustrated by the ORAC values, which are expressed as µM Trolox equivalents per 10 µM isoflavonoid (µM TE/10 µM). As shown in Table 3, several isoflavonoids appeared to have higher antioxidant activity than Trolox (higher than 20 µM TE), and the isoflavones exhibited higher activity than the isoflavanone and the isoflavans (Table 2). The isoflavanone 3*(R)*-dalparvin A (**18**) with the R7-OH, R5-OH, R2′-OMe, R4′-OH, and R5′,-OH substitutions gave the highest ORAC value (120 µM TE). The replacement of R4′-OH with -OMe, *i.e.*, 3*(R,S)-*dalparvin (**13**) (22 µM TE) reduced the ORAC values considerably. The absence of the free OH substitutions at Ring B rendered the isoflavanones completely inactive (*i.e.*, 3*(R,S)-*onogenin (**11**), 3*(S)*-sativanone (**10**), and 3*(RS)*-3′-O-methylviolanone (**9**)). Among the free OH substitutions, the positions of the R5′-OH and R3′-OH of Ring B appeared to be important. The presence of R2′-OMe, which showed a strong negative effect on the X/XO assay, did not have a significant effect on the ORAC assay. The subgroup of isoflavones also showed that the presence of R3′-OH (*i.e.*, khrinone C (**8**) (44 µM TE) and calycosin (**5**) (38 µM TE)) and presumably that of R5′-OH are important. In addition, the OH substitution at the R5 of Ring A is also important, as shown by the ORAC values of 27 µM TE for biochanin A (**2**) and 2.8 µM TE for formononetin (**1**). Both isoflavones differ from each other only by the absence of R5-OH in the latter molecule. The analysis of the isoflavans revealed that all of the isolates tested have R4′-OMe with mostly R3′-OH and thus showed non-significant differences in their ORAC values (30-40 µM TE). From these results, it was concluded that the R4′-OH substitution with either R3′-OH or R5′-OH and to a lesser extent R5-OH in the isoflavonoids promotes oxygen radical absorbance capacity and that the replacement of R4′-OH with -OMe significantly reduces the ORAC value.

**Table 3:** SAR of *D. parviflora* isoflavonoids based on the ORAC assay.

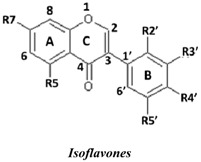	***No.***	***Isoflavones***	**R7**	**R5**	**R2′**	**R3′**	**R4′**	**R5′**	**(µM TE) ^†^**
	**8**	Khrinone C	OH	OH	OMe	OH	OMe	H	43.5 ± 3.2
	**5**	Calycosin	OH	H	H	OH	OMe	H	37.8 ± 1.2
	**3**	Genistein	OH	OH	H	H	OH	H	37.8 ± 4.5
	**6**	3′-O-Methylorobol	OH	OH	H	OMe	OH	H	35.7 ± 5.5
	**7**	Cajanin	OMe	OH	OH	H	OH	H	34.7 ± 2.2
	**4**	Khrinone B	OH	OH	OH	H	OMe	OH	34.2 ± 2.9
	**2**	Biochanin A	OH	OH	H	H	OMe	H	26.6 ± 1.3
	**1**	Formononetin	OH	H	H	H	OMe	H	2.8 ± 0.5
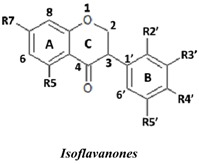	***No.***	***Isoflavones***	**R7**	**R5**	**R2′**	**R3′**	**R4′**	**R5′**	**(µM TE) ^†^**
	**18**	3(R)-Dalparvin A	OH	OH	OMe	H	OH	OH	120.3 ± 15.1
	**15**	3(R,S)-Kenusanone G	OH	OH	H	OH	OMe	H	42.1 ± 0.5
	**14**	Dalparvin B	OH	H	OH	OMe	OMe	H	33.4 ± 4.9
	**16**	3(R,S)-Violanone	OH	H	OMe	OH	OMe	H	31.1 ± 2.5
	**12**	3(R)-7,3′-Dihydroxy-4′-methoxyisoflavanone	OH	H	H	OH	OMe	H	28.4 ± 7.5
	**17**	3(S)-Secundiflorol H	OH	OH	OMe	OH	OMe	H	27.4 ± 7.7
	**13**	3(R,S)-Dalparvin	OH	H	OMe	H	OMe	OH	21.8 ± 1.5
	**11**	3(R,S)-Onogenin	OH	H	OMe	H	OCH_2_O	-
	**10**	3(S)-Sativanone	OH	H	OMe	H	OMe	H	-
	**9**	3(R,S)-3′-O-Methyl-violanone	OH	H	OMe	OMe	OMe	H	-
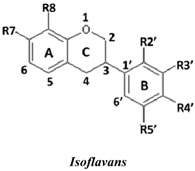	***No.***	***Isoflavones***	**R7**	**R5**	**R2′**	**R3′**	**R4′**	**R5′**	**(µM TE) ^†^**
	**21**	3(R)-Vestitol	OH	H	OH	H	OMe	H	40.1 ± 1.0
	**23**	3(R)(+)-Mucronulatol	OH	H	OMe	OH	OMe	H	39.8 ± 0.5
	**20**	3(R,S)-Duartin	OH	OMe	OMe	OH	OMe	H	34.2 ± 0.7
	**24**	3(R,S)-3′-Hydroxy-8-methoxyvestitol	OH	OMe	OH	OH	OMe	H	31.4 ± 2.7
	**22**	3(S)-8-Demethyl-duartin	OH	OH	OMe	OH	OMe	H	27.0 ± 1.9
	**19**	3(R,S)-Sativan	OH	H	OMe	H	OMe	H	24.8 ± 3.1

**^†^** Expressed as Trolox equivalents (TE, µM Trolox)/10 µM isoflavonoid; “-” no antioxidant activity was detected.

**Figure 1:**
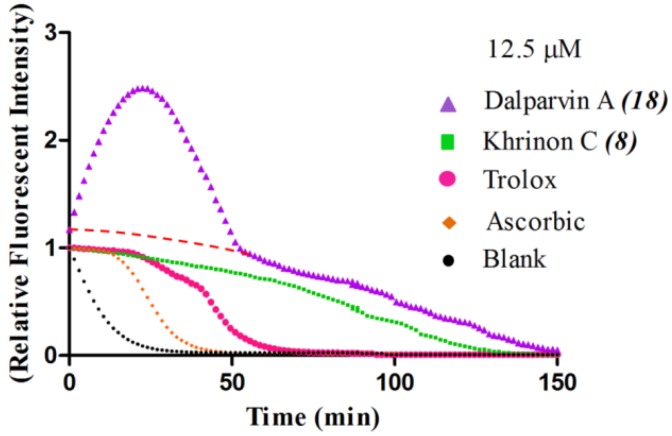
Fluorescence decay curve induced by AAPH. The signal curves of some of the tested compounds compared with that of the blank. The extrapolating decay line from the unusual experimental curve of dalparvin A (**18**) is indicated by the red dashed line (----).

The unusual higher ORAC value of 3*-(R)*-dalparvin A (**18**) (120 µM TE/10 µM) compared with the other isoflavonoids (20–40 µM TE/10 µM) led us study this compound further to determine whether there was a false-positive contribution to its ORAC value. The comparison of the fluorescent decay curves of 3*(R)*-dalparvin A (**18**), khrinone C (**8**), Trolox, and ascorbic acid (all at a concentration of 12.5 µM) revealed that 3*(R)*-dalparvin A (**18**) exhibits an unusual pattern in the fluorescent decay with an early increase in the area under the curve (Figure 1). It is likely that the observed increase in the curve is due to the thermal degradation of AAPH, which leads to the formation of peroxyl radicals that can oxidise the non-fluorescent 3*(R)*-dalparvin A (**18**) into an unknown fluorescent product over time. To cancel this effect, the fluorescent decay curve of the compound was normalised by extrapolating the decay line from the normal part of the curve (Figure 1). As a result, the presumably actual ORAC value of 3*(R)*-dalparvin A (**18**) was estimated to be 50 µM TE/10 µM, which is still higher than the ORAC values obtained for the other compound and thus does not affect the above SAR analysis of the set of the isoflavonoids.

### 2.3. SAR of D. parviflora Isoflavonoids Based on DPPH Radical Scavenging Activity

The bleaching of DPPH is one of the common methods used to evaluate the antioxidant properties of herbal extracts. The assay is based on measurement of the loss of DPPH colour at 515 nm after reaction with test compounds which is considered to be mainly based on an electron transfer reaction, and hydrogen-atom abstraction is a marginal reaction pathway [16]. The quantitative analysis of the DPPH radical scavenging activity of the *D. parviflora* isoflavonoids is shown in Table 4. It can be observed that several compounds exhibited high DPPH radical scavenging activity, particularly 3 *(R,S)*-3′-hydroxy-8-methoxyvestitol (**24**), 3*(R)*-dalparvin A (**18**), and khrinone C (**8**), which showed SC_50_ values of 38.7, 41.9, and 61.7 µM, respectively. The well-known antioxidant ascorbic acid was shown to have a SC_50_ value of 39.6 µM. The analysis of compounds belonging to the different subgroups but exhibiting the same substitution pattern showed that the subgroup of isoflavones, khrinone C (**8**), appeared to be more potent than the isoflavonones, 3*(S)*-secundiflorol H (**17**), and the isoflavan, 3*(S)*-8-demethylduartin (**22**) (Table 2).

In terms of SARs, it was found that, among the isoflavones, khrinone C (**8**), with R7-OH, R5-OH, R2′-OMe, R3′-OH, and R4′-OMe, showed the highest DPPH scavenging activity (SC_50_, 61.7 µM) (Table 3). The presence of R3′-OH (or OMe) or R5′-OH, similarly to the results found in the X/XO and ORAC assays, was also found to be important for DPPH scavenging activity. The absence of oxygen functionality at either of the two positions caused a complete loss of the activity, as was observed with genistein (**3**), biochanin A (**2**), and formononetin (**1**) (Table 3). The analysis of the isoflavanones also revealed the importance of R3′-OH or R5′-OH; in fact, the simultaneous presence of both R5-OH and R2′-OMe (or presumably R2′-OH) was particularly important in this subgroup. Similarly, the analysis of the isoflavans showed that the R3′-OH substitution and likely the R5′-OH substitution contribute to the activity.

**Table 4:** SAR of *D. parviflora* isoflavonoids based on the DPPH radical scavenging activity.

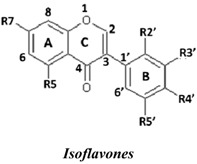	***No.***	***Isoflavones***	**R7**	**R5**	**R2′**	**R3′**	**R4′**	**R5′**	**SC_50_ (µM)**
	**8**	Khrinone C	OH	OH	OMe	OH	OMe	H	61.7 ± 4.5
	**7**	Cajanin	OMe	OH	OH	H	OH	H	70.8 ± 1.1
	**6**	3′-O-Methylorobol	OH	OH	H	OMe	OH	H	81.2 ± 14.1
	**5**	Calycosin	OH	H	H	OH	OMe	H	96.2 ± 2.8
	**4**	Khrinone B	OH	OH	OH	H	OMe	OH	133.6 ± 7.0
	**3**	Genistein	OH	OH	H	H	OH	H	-
	**2**	Biochanin A	OH	OH	H	H	OMe	H	-
	**1**	Formononetin	OH	H	H	H	OMe	H	-
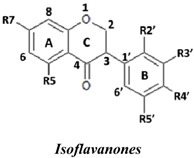	***No.***	***Isoflavones***	**R7**	**R5**	**R2′**	**R3′**	**R4′**	**R5′**	**SC_50_ (µM)**
	**18**	3(R)-Dalparvin A	OH	OH	OMe	H	OH	OH	41.9 ± 4.8
	**17**	3(3)-Secundiflorol H	OH	OH	OMe	OH	OMe	H	74.3 ± 4.2
	**12**	3(R)-7,3′-Dihydroxy-4′-methoxyisoflavanone	OH	H	H	OH	OMe	H	78.9 ± 1.1
	**13**	3(R,S)-Dalparvin	OH	H	OMe	H	OMe	OH	80.4 ± 1.3
	**16**	3(R,S)-Violanone	OH	H	OMe	OH	OMe	H	89.7 ± 1.7
	**15**	3(R,S)-Kenusanone G	OH	OH	H	OH	OMe	H	111.9 ± 4.7
	**14**	Dalparvin B	OH	H	OH	OMe	OMe	H	236.3± 9.8
	**11**	3(RS)-Onogenin	OH	H	OMe	H	OCH_2_O		-
	**10**	3(S)-Sativanone	OH	H	OMe	H	OMe	H	-
	**9**	3(R,S)-3′-O-Methyl-violanone	OH	H	OMe	OMe	OMe	H	-
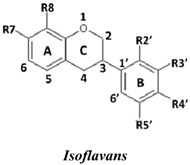	***No.***	***Isoflavones***	**R7**	**R5**	**R2′**	**R3′**	**R4′**	**R5′**	**SC_50_ (µM)**
	**24**	3(RS)-3′-Hydroxy-8-methoxyvestitol	OH	OMe	OH	OH	OMe	H	38.7 ± 3.0
	**23**	3(R)(+)-Mucronulatol	OH	H	OMe	OH	OMe	H	75.41 ± 3.2
	**22**	3(3)-8-Demethyl-duartin	OH	OH	OMe	OH	OMe	H	115.4 ± 4.3
	**21**	3(3)-Vestitol	OH	H	OH	H	OMe	H	204.1 ± 8.0
	**19**	3(R,S)-Sativan	OH	H	OMe	H	OMe	H	-
	**20**	3(R,S)-Duartin	OH	OMe	OMe	OH	OMe	H	-

“-” no antioxidant activity was detected.

Previous studies on the SAR of natural flavonoids based on the DPPH assay have indicated the importance of dihydroxy substitutions, *i.e.*, an *ortho-*dihydroxy structure (catechol structure) of the B-ring, which possesses electron-donating properties and is a radical target [17,18,19,20,21]. Our results also showed that 3*(R,S)-*3′-hydroxy-8-methoxyvestitol (**24**) and 3*(R)*-dalparvin A (**18**) with the *ortho*-catechol group in the B-ring gave the highest DPPH radical scavenging activity. On the contrary, the presence of steric obstruction due to the *ortho*-catechol group by O-methylation significantly affected the antioxidant activity. For example, the 2′-O-methylation of 3*(R,S)-*3′-hydroxy-8-methoxyvestitol (**24**) to 3*(R,S)-*duartin (**20**) markedly decreased the scavenging activity (Table 3). The O-methylation of the hydroxyl group on the other positions also caused a decrease in the DPPH radical scavenging activities, as was observed with formononetin (**1**), biochanin A (**2**), 3*(R,S)-*3′-O-methylviolanone (**9**), and 3*(R,S)-*sativan (**19**). The complete absence of DPPH scavenging activity found for genistein ***(3)*** is similar to the results reported previously [22,23,24], suggesting that the R4′-OH of Ring B is not important for radical scavenging activity with this assay method.

## 3. Experimental

### 3.1. Chemicals

1,1-Diphenyl-2-picrylhydrazyl (DPPH), (2,3-bis[2-methoxy-4-nitro-5-sulfophenyl]-2H-tetrazolium-5-carboxanilide (XTT), xanthine oxidase (EC 1.1.3.22, Grade IV), fluorescein, 6-hydroxy-2,5,7,8-tetramethyl-chroman-2-carboxylic acid (Trolox^TM^), and [2,2′-azobis(2-amidino-propane) dihydrochloride (AAPH) were purchased from Sigma Chemical Co. (St. Louis, MO, USA). The isoflavonoids of *D. parviflora* were isolated and identified as described previously [8,9].

### 3.2. Scavenging of Diphenyl-Picrylhydrazyl (DPPH) Radicals

According to the methods described by Blois [25], the free radical scavenging activity was measured using the DPPH assay with some modifications. Five microliters of different concentrations of each sample or 50% DMSO (final 1.25% DMSO concentration as a negative control) or 0.5 mg/mL ascorbic acid (as a positive control) were allowed to react with 195 µL of 100 µM DPPH methanolic solution in a 96-well microplate. The plate was then incubated at 37 °C for 30 min, and the absorbance was then measured at 515 nm (Beckman Coulter AD 200 UV/VIS Plate Reader, Brea, CA, USA). The DPPH radical reducing activity of the test sample was calculated using the following equation: Scavenging effect (%) = [(A_0_ − A_1_)/A_0_] × 100, where A_0_ is the absorbance of the control reaction, and A_1_ is the absorbance in the presence of the tested compound. The SC_50_ (concentration providing 50% inhibition) was calculated graphically using a calibration curve in the linear range by plotting the compound concentration versus the corresponding scavenging effect.

### 3.3. Inhibition of Superoxide Radical Formation by Xanthine/Xanthine Oxidase (X/XO Assay)

Reactive oxygen species were generated using the xanthine/xanthine oxidase system described by McCord and Fridovich [10]. Xanthine oxidase (XO) catalyses the univalent and divalent reduction of ground-state oxygen to generate both •O_2_^−^ and H_2_O_2_ and results in the oxidation of xanthine to uric acid. The radical formation was detected indirectly by measuring the rate of reduced XTT (2, 3-bis [2-methoxy-4-nitro-5-sulfophenyl]-2H-tetrazolium-5-carboxanilide, orange formazan dye). The mixture contained 120 µL of 50 mM NaHCO_3_ buffer (pH 9.4) with 1 mM EDTA, 20 µL of 0.5 mM hypoxanthine, and 20 µL of 0.25 mM XTT. Twenty microliters of either the sample or 50% DMSO (final 5.0% DMSO concentration as a negative control) was then added to the mixture. The reaction was initiated by the addition of 20 µL of 100 mU/mL XO, and the production of reduced XTT was then kinetically determined at 475 nm every 30 s over a period of 10 min (Beckman Coulter AD 200 UV/VIS Plate Reader). The percentage of reduced XTT at steady state was calculated, and these values were plotted against the concentrations of the test compounds. The results are expressed as the concentration of the test compounds that scavenged 50% of the free radicals (SC_50_).

### 3.4. Measurement of Oxygen Radical Absorbance Capacity (ORAC)

The oxygen radical absorbance capacity (ORAC) assay measures the antioxidant inhibition of peroxyl radical-induced oxidation and thus reflects the classical chain-breaking antioxidant capacity activity by H atom transfer. The samples were assayed using previously described methods with some minor modifications [26,27]. The assay was conducted in black-walled 96-well plates. Each well had a final volume of 200 µL. The reaction mixture, which contained 160 µL of 10 nM fluorescein solution in 10 mM phosphate buffer (pH 7.4), was added to 20 µL of the sample or 20 µL of 50% DMSO (final 5.0% DMSO concentration as a blank), and the mixture was pre-incubated at 37 °C for 10 min. To start the reaction, 20 µL of 240 mM AAPH, a peroxyl radical generator, was added to the pre-incubated mixture. A change in the intensity of the fluorescent probe caused by free radicals was then monitored at 37 °C every 90 sec for a period of 60 min using a fluorescent microplate reader (Beckman Coulter DTX880 Multimode Detector) at the excitation and emission wavelengths of 485 and 530 nm, respectively. In parallel, 50, 25, 12.5, and 6.25 µM Trolox™, a water-soluble vitamin E analogue, was used as a standard. The areas under the fluorescence decay curves (AUC) were analysed using the GraphPad Prism software and subtracted by the AUC of the blank to obtain the net AUC. The graphs of the net AUC and Trolox™ standard concentrations were plotted, and the μmole Trolox equivalents were then calculated.

## 4. Conclusions

A set of 24 isoflavonoids isolated from the heartwood of *D. parviflora* shows diversified structures that can be grouped into three subgroups, namely isoflavones, isoflavanones, and isoflavans, and is thus ideal for an SAR study. Due to the scarcity of information in the literature, this study aimed to investigate the structure and antioxidant activity relationships of the isoflavonoids using three common *in vitro* methods with different working principles: the xanthine oxidase free radical generating system (X/XO assay), the oxygen radical absorbance capacity assay (ORAC assay), and the DPPH radical scavenging activity (DPPH assay). The results showed that each assay type gave relatively different preferences of antioxidant activities among the subgroups: the X/XO assay showed isoflavones > isoflavans > isoflavanones, the ORAC assay showed isoflavones > isoflavanones ~ isoflavans, and the DPPH showed isoflavones > isoflavanones > isoflavans. The intra-subgroup analysis showed that the additional presence of an OH group in Ring B at either R3′ or R5′ greatly increased the antioxidant activities of all three isoflavonoid subgroups compared with the basic common structure of R7-OH in Ring A and R4′-OH (or -OMe) in Ring B. However, each assay type also showed its own pattern of SAR preference among the three subgroups. Specifically, the X/XO assay showed that the R2′-OMe substitution in the isoflavanones and isoflavans exhibited a strong negative effect, whereas the ORAC assay showed the importance of non-methylated free OH substitutions in the Ring B of all three subgroups and the important of the presence of R5-OH in Ring A in the isoflavones, and the DPPH assay showed the relatively strong positive effect of the co-presence of both the R5-OH of Ring A and the R2′-OMe of Ring B in the isoflavanone subgroup. These results from the analysis of the structure and antioxidant activity relationships of isoflavonoids will be useful for the design of isoflavonoids possessing antioxidant activities, which will prove beneficial for health.

## Abbreviations

SC_50_: (50% radical scavenging concentration)
ORAC: oxygen radical absorbance capacity
AUC: areas under the fluorescence decay curves
AAPH: 2,2′-azobis(2-amidinopropane) dihydrochloride
